# Splenic abscess secondary to COVID-19 acute infection: A case report and literature review

**DOI:** 10.1097/MD.0000000000039194

**Published:** 2024-08-02

**Authors:** Wei Wang, Wen-Hui Zhai, Ying Zhang, Li Tao, Yun Li, Tong-xue Jiang, Jin-ping Zhang

**Affiliations:** a Emergency Department, 305 Hospital of People's Liberation Army, Beijing, People’s Republic of China; b Outpatient Department, 305 Hospital of People's Liberation Army, Beijing, People’s Republic of China; c Medical Department, 305 Hospital of People's Liberation Army, Beijing, People’s Republic of China.

**Keywords:** abscess, case report, COVID-19, lung infection, spleen

## Abstract

**Rationale:**

Splenic abscess is relatively rare in clinical practice as an invasive disease. However, during the continuous prevalence of coronavirus disease 2019 (COVID-19), the incidence rate of splenic abscess showed an upward trend. However, because the etiology of splenic abscess is not specific, it is easy to be covered by the respiratory symptoms of COVID-19, resulting in omission or delay in diagnosis. If splenic abscesses cannot be treated in a timely manner, the mortality rate can reach 100%. Therefore, it is important to fully understand the correlation between COVID-19 and the development of splenic abscesses.

**Patient concerns:**

A female patient, 71 years of age, was admitted to our hospital because of cough and sputum for 1 week and fever for 2 days. According to the positive results of novel coronavirus nucleic acid and chest computed tomography, novel coronavirus pneumonia was diagnosed. On the 4th day after treatment, abdominal distension and vomiting were observed. Abdominal ultrasound indicated splenomegaly and mixed echo masses in the spleen and abdominal computed tomography indicated 2 new round low-density lesions were found in the spleen.

**Diagnoses:**

The patient was diagnosed with secondary splenic abscess after COVID-19 infection.

**Interventions:**

The patient and her family members refused to undergo ultrasound-guided splenic puncture drainage and splenectomy. In terms of treatment, she was given meropenem combined with vancomycin to continue anti-infection treatment.

**Outcomes:**

The patient’s body temperature and infection indicators gradually increased, and the scope of splenic abscess continued to expand. The infection worsened and progressed to septic shock. The patient abandoned rescue drugs and invasive treatment, and died on the 9th day after admission.

**Lessons:**

This case introduces the clinical characteristics of secondary splenic abscess caused by COVID-19 from the aspects of etiology, disease course, clinical manifestations, auxiliary examinations, and treatment methods. The focus is on improving the understanding of clinical doctors about secondary splenic abscesses caused by COVID-19, providing reference for early diagnosis and timely treatment.

## 1. Introduction

Coronavirus disease 2019 (COVID-19) is still spreading worldwide, and the number of severe cases and deaths caused by COVID-19 remains high. In most cases, COVID-19 presents primarily with respiratory symptoms, and a few patients may involve or develop secondary abdominal organ lesions. Among them, cases of secondary splenic abscess are even rarer, and often misdiagnosed due to the lack of specific clinical manifestations. Given the ongoing prevalence of COVID-19, accurate diagnosis and timely treatment of splenic abscesses are crucial. This case has been reported in line with the SCARE 2020 criteria for case reports.^[1]^

## 2. Case presentation

A 71-year-old female with a history of chronic lymphocytic leukemia, hypertension, and coronary heart disease presented to the emergency department on October 13 with cough and sputum for 1 week and fever for 2 days. At the beginning of the disease, the patient’s body temperature was up to 38.3°C, accompanied by chest tightness, shortness of breath, listlessness, and no gastrointestinal symptoms such as abdominal discomfort and vomiting. The patient denied a recent history of abdominal trauma, long-term oral hormone medication, denial of eating raw meat and lettuce, and had not been vaccinated against COVID-19.

Physical examination showed moist rales in the lower left lung, no abdominal tenderness, and no abdominal organ enlargement. The infection indicators upon admission were as follows: white blood cell (WBC): 3.0 × 10^9^/L (reference value: 3.5–9.5 × 10^9^/L), C-reactive protein: 10.1 mg/dL (reference value: <0.8 mg/dL), interleukin-6: 59.71 pg/mL (reference value: <7 pg/mL), procalcitonin: 0.092 ng/mL (reference value: <0.25 ng/mL). Chest computed tomography (CT) scan showed multiple ground glass shadows and consolidation in both lungs. COVID-19 was diagnosed based on a positive nucleic acid test, and molnupiravir, dexamethasone, enoxaparin sodium, and moxifloxacin were given. After treatment, the patient did not have fever again, and cough and expectoration were alleviated.

**Figure 1.**
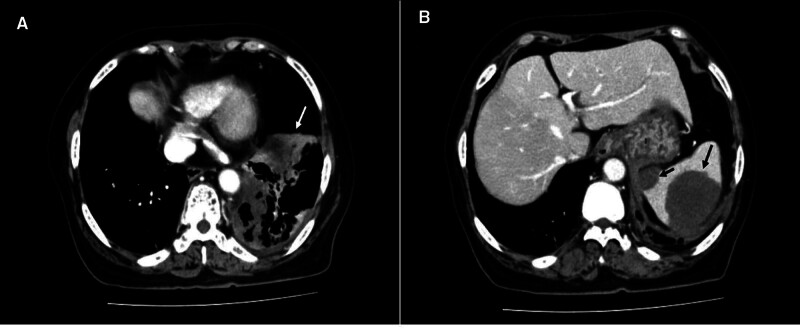
Enhanced CT of the abdomen. (A) Lung infection (white arrow). (B) Splenic abscess (black arrow). CT = computed tomography.

Day 5 (October 17), the patient had slight abdominal distention, accompanied by vomiting and no abdominal pain. Upon examination, the abdomen was flat, no intestinal pattern and peristaltic waves were seen, the abdomen was soft, the left upper quadrant was mildly tender, there was no muscle tension and rebound tenderness, the liver and spleen were not palpated under the costal level, the tympanum sound was percussion, and the bowel sounds were normal. Blood tests showed no abnormal changes in infection indicators, and amylase and lipase values were normal. Abdominal ultrasound showed splenomegaly and mixed echogenic masses in the spleen. Abdominal enhanced CT showed aggravation of left lower lung lesions (Fig. 1A), and 2 new round low-density lesions (CT values 33–39 HU) were found in the spleen, the larger one was 5.1 × 4.3 cm, and the enhancement of the lesions at each stage was not obvious (Fig. 1B). Of note, a CT scan from 6 days prior showed a normal spleen. Splenic abscess was diagnosed based on clinical symptoms and imaging examination. The POSSUM score predicted a complication rate of 54.24% and a mortality rate of 10.91%. Ultrasound-guided transsplenic puncture drainage was considered, but the patient and their family refused. Meropenem and vancomycin were given for anti-infection, and continued comprehensive treatment for COVID-19.

From the seventh day (October 19), the patient’s body temperature increased again and continued (Table 1), C-reactive protein, interleukin-6, and procalcitonin continued to rise, and WBC continued to decrease (Table 1), indicating an aggravation of infection. Transesophageal echocardiography showed no evidence of endocarditis. Perfecting 3 sets of blood cultures resulted in no organisms growing. Day 8 (October 20), the patient developed drowsiness with increased respiratory rate and progressed to sepsis. The follow-up abdominal ultrasound showed that 2 abscesses were fused and the risk of rupture was extremely high. The family still refused to intervene with a puncture procedure. Day 9 (October 21), the patient continued to have high fever with coma (Glasgow Coma score was 6) and secondary acute respiratory distress syndrome, and the abdominal ultrasound showed that the abscess continued to increase with a small amount of fluid around the spleen. Day 10 (October 22), the patient’s mean arterial pressure decreased and progressed to septic shock, and the family abandoned rescue drugs and invasive treatment measures, resulting in death on the same day (Table 1).

**Table 1:** Summary of cases (body temperature, indicators of infection, and abscess size).

Time of disease progression	Maximum body temperature (°C)	WBC (×10^9^/L)	CRP (mg/dL)	IL-6 (pg/mL)	PCT (ng/mL)	Abscess size (cm)
October 13	36.2	3.0	10.1	59.71	0.092	—
October 16	37.6	5.2	13.7	205	0.1	—
October 17	36.9	5.1	14.4	—	—	4.3 × 3.8
October 18	36.8	—	—	—	—	—
October 19	38.7	4.4	18.8	457	0.26	5.5 × 4.6
October 20	39.3	—	—	—	—	7.7 × 5.6
October 21	40.0	2.2	23.1	945.5	2.2	9.0 × 7.0
October 22	40.1	—	—	—	—	—

CRP = C-reactive protein, CT = computed tomography, IL-6 = interleukin-6, PCT = procalcitonin, WBC = white blood cell.

## 3. Discussion and conclusions

As an invasive disease, splenic abscess is relatively rare in clinical practice, with an incidence of approximately 0.07% to 0.2%.^[2,3]^ During the current COVID-19 epidemic, the incidence rate of splenic abscesses has increased significantly,^[4]^ but because the etiology of splenic abscesses is not specific, it is easy to be covered by the respiratory symptoms of COVID-19, resulting in omission or delay in diagnosis, resulting in higher mortality. Therefore, it is important to fully understand the correlation between COVID-19 and the development of splenic abscesses.

Typical causes of splenic abscess are classified into 4 categories, including metastatic spread from septic foci, which is the most common and often involves the transfer of pathogens from lung infections, bacterial endocarditis, typhoid fever, malaria, and other diseases to the spleen through blood, and this kind of splenic abscess is mostly combined with abscesses of other organs; direct spread of adjacent organ infections, such as gastrointestinal tumors, gastrointestinal perforation, left thoracic infection, etc; traumatic factors: such as ligation of splenic artery branches, splenic trauma, or infarction leading to local hematoma and subsequent abscess formation; immune dysfunction status, including cancer, diabetes, alcoholism, immunosuppressive therapy, and cirrhosis.^[4,5]^

Regarding the cause of splenic abscess formation caused by COVID-19, AlZarooni et al^[4]^ and Khan et al^[6]^ believe that it is related to inflammatory response, viral virulence, and splenic artery thrombosis.^[7]^ It has also been reported in the literature that patients with COVID-19 are at risk of splenic hemorrhage and hematoma formation when they receive high-dose anticoagulation, which can lead to secondary abscess formation.^[8]^ In this case, in addition to the 2 possible factors mentioned above, the patient had a history of chronic lymphocytic leukemia, which weakened the chemotaxis and phagocytic function of cells, resulting in a decrease in the patient’s immune system. Additionally, immunosuppression could be a triggering factor caused by hormones in COVID-19 treatment. Moreover, due to the close relationship between the lesion in the left lower lobe of the lung and splenic abscess in imaging, no signs of abscess were found in other organs, and multiple blood cultures were negative, we highly suspected that the direct spread of the infection lesion in the left lower lung leaded to the formation of splenic abscess.

In the course of splenic abscess secondary to COVID-19, some studies speculated that the direct cause of this complication may be the immunosuppressive state after a long course of COVID-19.^[9,10]^ However, in recent reports, AlZarooni et al^[4]^ reported that 2 of the 3 patients with splenic abscesses secondary to COVID-19 occurred in the acute phase of infection, and 1 occurred approximately 1 month after infection, Cairl and Sharp^[2]^ reported that a splenic abscess occurred several weeks after COVID-19 infection. In this case, the splenic abscess occurred on the 11th day after COVID-19 infection. From the reviewed literature and our case, it can be assumed that the formation of a splenic abscess can occur at any stage after COVID-19 infection.

In terms of clinical manifestations, the triad of fever, left upper quadrant abdominal pain, and leukocytosis has been suggested to be associated with splenic abscess, but it is reportedly only present in one-third of cases.^[3,11,12]^ However, based on the understanding of COVID-19, we believe that in the case of new coronavirus infection, especially in the acute phase of infection, due to the superposition of fever symptoms, abdominal symptoms are masked by respiratory symptoms, and the virus invasion leads to low WBC, so the probability of splenic abscess triad will be less. The patient reported in this article only presented with mild abdominal distension with vomiting, the body temperature did not rise in the 2 days prior to diagnosis, and the WBC did not rise but decreased throughout the course of the disease, which further confirmed our hypothesis.

Due to the nonspecific clinical manifestations of splenic abscess, timely imaging examination is necessary when suspicious symptoms appear. On imaging studies, CT and ultrasound are both sensitive for splenic abscesses, and CT is considered the gold standard for diagnosing.^[3]^ Imaging findings often include splenomegaly, intrasplenic gas, splenic cystic lesions, or progressive enlargement of splenic lesions. Based on clinical symptoms and imaging, the diagnostic rate of splenic abscess is approximately 86.7%.^[13]^ Once splenic abscess is diagnosed, immediate treatment is necessary, otherwise, the mortality rate can reach 100%.^[14]^ The treatment of splenic abscess adopts a combination of systemic medication and local lesion management, but the optimal treatment strategy is still controversial. Usually, anti-infection treatment is given based on drug sensitivity analysis, while paying attention to supportive therapy. If the abscess is large and single, percutaneous drainage can be performed. If multiple abscesses are present, splenectomy can be directly administered for treatment. Recently, percutaneous drainage has replaced traditional splenectomy as the preferred initial intervention for splenic abscess,^[15]^ preserving important immune functions of the spleen and reducing the incidence of treatment complications (splenectomy vs percutaneous drainage: 26% vs 10%).^[16]^ In the case of spleen abscess secondary to COVID-19, in addition to selecting appropriate splenic abscess interventions, active treatment of COVID-19 is also very important, and uncontrolled new crown infection can increase spleen abscess-related mortality and eventually death from septic shock, multi-organ failure, etc.

In this case, due to the existence of chronic lymphoblastic leukemia, the patient’s own immune function is poor, which is very likely to lead to COVID-19 infection. At the same time, it will seriously affect the treatment effect, increase the incidence of complications and critical diseases, and increase the risk of death. The patient found spleen abscess on the 9th day after COVID-19 infection, and the specificity of clinical symptoms was low. According to the anatomical relationship of the focus, the lung infection focus directly spread to invade the adjacent spleen, and the disease continued to progress under the condition of active anti-infection. Due to the lack of invasive intervention and the lack of patient’s own immunity, it finally progressed to septic shock in a short time, and then died.

The spleen abscess in this case was closely related to COVID-19 infection. There were clear and typical signs on imaging, and the spleen abscess was closely related to lung lesions. However, the absence of invasive intervention could not further determine the clear pathophysiological association between the diseases from histopathological studies. Additionally, due to the limited number of cases, it may affect the accuracy of some conclusions and have certain limitations. In the future, we will continue to pay attention to such cases for further summary and analysis. This article focuses on improving clinicians’ understanding of splenic abscess secondary to COVID-19 and providing a reference for early diagnosis and timely treatment.

## Acknowledgments

Thanks are due to Doctor Mei-yu Deng for valuable discussion.

## Author contributions

**Investigation:** Wen-Hui Zhai, Yun Li.

**Supervision:** Wen-Hui Zhai, Jin-ping Zhang, Tong-xue Jiang.

**Writing—original draft:** Wei Wang, Ying Zhang, Li Tao.

**Writing—review & editing:** Wei Wang, Jin-ping Zhang Zhang.

## References

[1] Agha RA Franchi T Sohrabi C Mathew G Kerwan A; SCARE Group. The SCARE 2020 guideline: updating consensus Surgical CAse REport (SCARE) guidelines. Int J Surg. 2020;84:226–30. 33181358 10.1016/j.ijsu.2020.10.034

[2] Cairl N Sharp V. Splenic abscess secondary to COVID-19 infection - a case report. Int J Surg Case Rep. 2022;101:107807. 36455347 10.1016/j.ijscr.2022.107807 PMC9705012

[3] Lotfollahzadeh S Mathew G Zemaitis MR. Splenic abscess. In: StatPearls. Treasure Island (FL): StatPearls Publishing; 2023. https://www.ncbi.nlm.nih.gov/books/NBK519546/. Accessed June, 2023. 30137831

[4] AlZarooni N AlBaroudi A AlOzaibi L AlZoabi O. Splenic abscess as a possible sequela of COVID-19: a case series. Ann Saudi Med. 2021;41:307–11. 34618603 10.5144/0256-4947.2021.307 PMC8497008

[5] Neligan P. Anesthesia and Uncommon, Diseases. 6th ed. 2012:53S–70S.

[6] Khan IH Savarimuthu S Leung MST Harky A. The need to manage the risk of thromboembolism in COVID-19 patients. J Vasc Surg. 2020;72:799–804. 32417304 10.1016/j.jvs.2020.05.015 PMC7224653

[7] de Roquetaillade C Chousterman BG Tomasoni D. Unusual arterial thrombotic events in Covid-19 patients. Int J Cardiol. 2021;323:281–4. 32918938 10.1016/j.ijcard.2020.08.103 PMC7481127

[8] Sherman Scott C Weber Joseph M Schindlbeck Michael A Patwari Rahul G. Clinical Emergency Medicine, Lange Medical Books. 2014.

[9] Li G Gao L Zhou J. Management of splenic abscess after splenic arterial embolization in severe acute pancreatitis: a 5-year single-center experience. Gastroenterol Res Pract. 2019;2019:6069179–5. 31354807 10.1155/2019/6069179 PMC6636444

[10] Rory Smoot L. Mark Truty J. David M. Nagorney, Shackelford’s Surgery of the Alimentary Tract, 2 Volume Set (8th ed., 2019;1635–1653.

[11] Lee WS Choi TS Kim KK. Splenic abscess: a single institution study and review of the literature. Yonsei Med J. 2011;52:288–92. 21319348 10.3349/ymj.2011.52.2.288 PMC3051211

[12] Alvi AR Kulsoom S Shamsi G. Splenic abscess: outcome and prognostic factors. J Coll Physicians Surg Pak. 2008;18:740–3. 19032885

[13] Ng KK Lee TY Wan YL. Splenic abscess: diagnosis and management. Hepatogastroenterology. 2002;49:567–71. 11995499

[14] Colmeneroa JD Queipo-Ortuñob MI Regueraa JM. Chronic hepatosplenic abscesses in brucellosis. clinico-therapeutic features and molecular diagnostic approach. Diagn Microbiol Infect Dis. 2002;3:159–67. 10.1016/s0732-8893(01)00344-3 11929686

[15] Al-Ozaibi LS Alshaikh MO Makhdoom M Alzoabi OM Busharar HA Keloth TR. Splenic abscess: an unusual presentation of COVID-19. Dubai Med J. 2020;3:115–8.

[16] Gutama B Wothe JK Xiao M Hackman D Chu H Rickard J. Splenectomy versus imaging-guided percutaneous drainage for splenic abscess: a systematic review and meta-analysis. Surgical Infect. 2022;23:417–29. 10.1089/sur.2022.072 PMC9208856 35612434

